# Tubal ligation and endometrial Cancer risk: a global systematic review and meta-analysis

**DOI:** 10.1186/s12885-019-6174-3

**Published:** 2019-10-11

**Authors:** Laleh Loghmani, Nafise Saedi, Reza Omani-Samani, Saeid Safiri, Mahdi Sepidarkish, Saman Maroufizadeh, Arezoo Esmailzadeh, Maryam Shokrpour, Esmaeil Khedmati Morasae, Amir Almasi-Hashiani

**Affiliations:** 1 Department of Nursing, Faculty of Nursing and Midwifery, Bam University of Medical Sciences, Bam, Iran; 2 Department of Gynecologic Oncology, Tehran University of Medical Sciences, Tehran, Iran; 3 Department of Medical Ethics and Law, Reproductive Biomedicine Research Center, Royan Institute for Reproductive Biomedicine, ACECR, Tehran, Iran; 4 Aging Research Institute, Tabriz University of Medical Sciences, Tabriz, Iran; 5 Department of Community Medicine, School of Medicine, Tabriz University of Medical Sciences, Tabriz, Iran; 6 Department of Biostatistics and Epidemiology, Babol University of Medical Sciences, Babol, Iran; 7 School of Nursing and Midwifery, Guilan University of Medical Sciences, Rasht, Iran; 8 Department of Obstetrics and Gynecology, Baqiyatallah University of Medical Sciences, Tehran, Iran; 9 Department of Obstetrics and Gynecology, Arak University of Medical Sciences, Arak, Iran; 10 Institute of Psychology, Health, and Society, Department of Health Services Research, University of Liverpool, Liverpool, UK; 11 Department of Epidemiology, School of Health, Arak University of Medical Sciences, Arak, Iran; 12 Traditional and Complementary Medicine Research Center, Arak University of Medical Sciences, Arak, Iran

**Keywords:** Endometrial neoplasms, Tubal sterilization, Tubal ligation, Meta-analysis

## Abstract

**Background:**

Studies on relationship between tubal ligation and endometrial cancer have led to contradictory findings. In several studies, however, a reduced endometrial cancer risk was suggested following tubal ligation. Therefore, a systematic review and meta-analysis was conducted to examine the relationship between tubal ligation and endometrial cancer risk.

**Methods:**

In this systematic review and meta-analysis, PubMed/Medline, Web of Science, Scopus, Embase, and Google Scholar were searched for relevant studies published up to May 30th, 2018. We compared endometrial cancer risk in women with and without tubal ligation in retrieved studies.

**Results:**

Two hundred nine studies were initially retrieved from the data bases. After exclusion of duplicates and studies which did not meet inclusion criteria, ten cohort and case-control studies, including 6,773,066 cases, were entered into the quantitative meta-analysis. There was 0.90% agreement between two researchers who searched and retrieved the studies. The summary OR (SOR) was reported using a random effect model. Begg’s test suggested that there was no publication bias, but a considerable heterogeneity was observed (I^2^ = 95.4%, *P* = 0.001). We pooled the raw number of tables cells (i.e. a, b, c, and d) of eight studies. The SOR suggested that tubal ligation was significantly associated with a lower risk of endometrial cancer (SOR = 0.577, 95% CI = 0.420–0.792). Also, given the rare nature of endometrial cancer (< 5%), different effect sizes were considered as comparable measures of risk. Therefore we pooled ten studies and SOR of these studies revealed that tubal ligation was significantly associated with a lower risk of endometrial cancer (SOR = 0.696, 95% CI = 0.425–0.966). Besides that, we pooled eight studies in which adjusted effect sizes were reported and a subsequent analysis revealed that the summary estimate of adjusted odds ratio (SAOR) was significant (SAOR = 0.862, 95% CI = 0.698–1.026).

**Conclusions:**

This study revealed a protective effect of tubal ligation on endometrial cancer risk (approximately 42% lower risk of cancer). It is recommended that studies should be designed to reveal mechanisms of this relationship.

## Highlights

- Concerning the relationship between tubal ligation and endometrial cancer, there are contradictory results.
- In this study we systematically reviewed all previous published studies and then we performed a meta-analysis.
- This study revealed a protective effect of tubal ligation on endometrial cancer risk by approximately 42%.

## Background

According to GLOBOCAN worldwide cancer incidence and mortality report, there are annually 14.1 million new cases and 8.1 million deaths due to cancers [1]. According to that report, endometrial cancer is the most frequent gynecological malignancy and the sixth most frequent malignancy among women in the world [2, 3]. In 2012, 320,000 new cases of endometrial cancer were reported worldwide [1]. Standard methods of endometrial cancer treatment include hysterectomy, bilateral salpingo-oophorectomy, and staging. Five-year survival rate varies between 74 and 91% in cases with no metastases [3].

Tubal ligation, known as female sterilization, is a permanent contraceptive method with more than 99% effectiveness. According to 2015 World Health Organization Report, this method was used by 19% of women worldwide [4]. However, its prevalence varies extensively around the world with a prevalence of 22% in United States and lower than 10% in Europe [2]. There are also some reports of dwindling interest in this contraceptive method. For instance, Chan and Westhoff suggested that number of tubal sterilization cases has been declining in recent years in United States [5]. Nowadays, around 700,000 bilateral tubal ligation (BTL) sterilization operations are annually carried out in United States and 11 million US women, in overall, use BTL [6] (it was 10.s million in 2002 [7]). More than 190 million couples in the world also use surgical sterilizations for contraception [6].

There is some evidence regarding the role of salpingectomy in reducing the risk of epithelial ovarian cancer [8, 9]. Bilateral salpingo-oophorectomy decreases the risk of ovarian cancer, but increases the risk of other cancers, cardiovascular disease, and all-cause mortality [10]. Not only does Salpingectomy during benign gynecological surgery, such as hysterectomy, reduce the risk of ovarian cancer, it is also a cost-effective process which does not influence ovarian function [11].

Several studies have concluded that tubal ligation is associated with the following outcomes: a reduction in risk and mortality of endometrial cancer [2, 12], a less chance of endometrial cancer diagnosis at advanced stages [13], lower positive peritoneal cytology, recurrence rate, and metastatic spread of non-endometrioid endometrial carcinoma [14]. On the contrary, several other studies have indicated that there is no such a link between tubal ligation and risk of endometrial cancer [15–17], pointing to a contradiction in our knowledge about this cancer. Falconer et al. [2] postulated that this contradiction can be attributed to methodological limitations and insufficient sample sizes in published studies. Motivated to solve the contradiction, in this study we systematically reviewed all the previously published studies and performed a meta-analysis to determine the association between tubal ligation and endometrial cancer risk.

## Methods

### Search strategy and study selection

In this systematic review and meta-analysis, we followed the standard guideline of “Preferred Reporting Items for Systematic Reviews and Meta-Analyses (PRISMA)” [18] and the “Cochrane Handbook for Systematic Reviews of Interventions” [19] to conduct the review and analysis. Studies, published before May 2018, in which the association between tubal ligation and endometrial cancer was investigated were included into our review pool. To retrieve the relevant articles, numerous databases, such as PubMed/Medline, Web of Science, Scopus, Embase, and Google Scholar, were searched. Informed by medical subject headings (MeSH), the following keywords were used to search the databases: “Endometrial Neoplasms”, “Endometrial Cancer”, “Endometrium Cancer”, “Endometrial Carcinoma”, “Endometrium Carcinoma”, “Tubal ligation”, “Bilateral tubal ligation”, “Sterilization, Tubal”, “Tubal Sterilization” and “Tubal Occlusion”. The search was performed by corresponding author and was re-checked by an epidemiologist (MS1). It should be mentioned that all kinds of tubal ligation including mono-lateral or bi-lateral salpingectomy, tubal coagulation with or without cut, and tubal ligation by stitches with or without cut were included into the meta-analysis.

### Inclusion and exclusion criteria

Studies published in English, within the defined timeframe, with a non-randomized design (i.e. case-control, cohort, registries, and cross-sectional studies) were included in the study. Review articles, letters to the editor, and commentaries were excluded. Retrieved records from the databases were entered into the Endnote reference manager (version X7) in order to categorize, manage, remove duplicates, and record titles, abstracts, and full-texts. Finally, titles and abstracts of the articles were evaluated and after removal of unrelated articles, full texts of the remaining articles were studied. In the cases of relevant articles in which the required data were not reported, an e-mail was sent to their corresponding author and the required information was collected. In order to prevent from missing the related articles the references of retrieved articles were also manually searched. All the above-mentioned steps were carried out by two independent researchers (SM and MS1) and their disagreements were resolved in consultation with other research team members.

### Data extraction

The retrieved full text of the articles was independently evaluated by two researchers (AAH and SS) and consulted with other research team members in case of disagreement. There was 90% agreement between two independent researchers and they strongly disagreed on one study. Information about authorship, date of publication, sample size, study design, number of endometrial cancer cases in each group (with and without tubal ligation), place of study, and studied population were extracted from the papers. These data were entered into an Excel sheet for preparation and cleaning. Raw numbers of the tables (a, b, c and d cells) as well as crude and adjusted effect sizes were also extracted from the papers.

### Risk of bias

To check for risk of bias in papers, Newcastle-Ottawa assessment scale adapted for case-control and cohort studies (two separate checklists) was used [20]. The scores of this checklist range from 0 to 9, categorized into three following levels: more than 6 as high, 3 to 6 as moderate, and less than 3 as low quality.

### Statistical analysis

Cochran’s Q test (with *P*-value < 0.10) and I-square statistic were used to check for heterogeneity across the studies (I-square more than 50% was considered as substantial heterogeneity). In cases where there was significant heterogeneity in results of primary studies, the source of heterogeneity was investigated using a meta-regression method. By this method some available and potential sources of heterogeneity such as date of publication, sample size, study quality scores, and study design were checked by “metareg” command in Stata [21].

To explore the between-study variance, Tau-squared statistic and to explore the publication bias, Egger’s linear regression was used. In cases where publication bias was present, the trim and fill method was used. The subgroup analysis was performed for date of publication, sample size, study quality scores, and study design. Because of the presence of heterogeneity problem, random-effects meta-analysis model was conducted to examine the relationship between tubal ligation and endometrial cancer risk. In this random-effects analysis, Odds Ratios (OR) were summarized and a summary OR (SOR) was reported to examine the relationship. Moreover, a sensitivity analysis was performed when required. All analyses were done using Stata software version 14 (Stata Corp, College Station, TX).

## Results

### Study selection and study characteristics

**Fig. 1:**
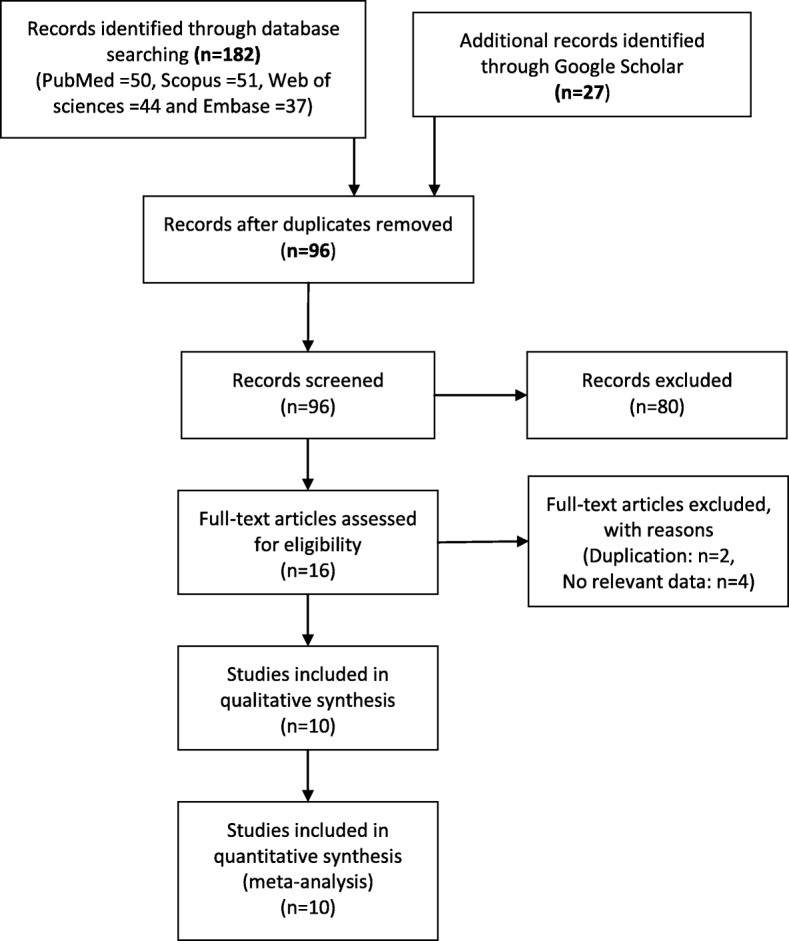
Flow diagram of the literature search for studies included in meta-analysis

**Fig. 2:**
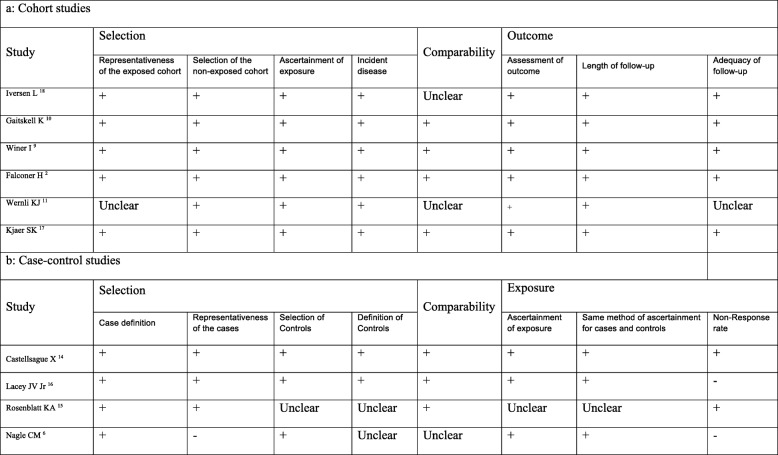
Quality assessment of included studies

A flow diagram of the literature search for studies that were included into the meta-analysis is presented in Fig. 1. Using the search strategy mentioned above, we managed to retrieve 209 studies (PubMed/Medline: 50, Scopus: 51, Web of science: 44, Embase: 37 and Google Scholar: 27 studies). After removal of duplicates, 96 articles finally remained for the analysis. After screening the titles and abstracts of these papers, 80 articles that did not match the inclusion criteria were omitted. Full texts of the remaining 16 articles were studied and 6 articles were excluded (2 studies due to duplication and 4 studies due to irrelevant data). Finally, data of 10 remaining papers [2, 12, 15–17, 22–26] was extracted and entered into the meta-analysis. Results of the Newcastle-Ottawa assessment scale for those 10 studies are presented in Fig. 2.

The specifications of the extracted studies are reported in Table 1. The total sample size of the include studies was 6,773,066 cases, coming from 4 case-control and 6 cohort studies. According to Table 1, the included studies were conducted between 1996 and 2018. The sample size of studies ranged from 701 [24] in the US to 5,385,186 [2] in Switzerland. Most studies were from the US with 3 studies, followed by the UK with two studies. These studies were carried out in different populations (i.e. women who had a tubal sterilization, women employed in the textile industry, cancer registries data, women who were using oral contraceptives, etc.).

**Table 1:** Characteristics of the primary studies included in the meta-analysis

First author	DOP	SS	Design	Country	Population	Study quality*	# of cases in TL+	# of TL+	# of cases in TL-	# of TL-	TES	ES	UL	LL	AES	UL	LL	Adjusted for:
Castellsague X ^14^	1996	3634	Case control	USA	20 to 54 years	High	53	671	383	2963	OR	0.58	0.43	0.78	0.87	0.63	1.2	Age and Parity
Lacey JV Jr. ^16^	2000	701	Case control	USA	20 to 74 years	High	47	87	357	614	OR	0.9	0.6	1.4	1.4	0.8	2.3	Age, parity, and OC usage
Kjaer SK ^17^	2004	65,232	Cohort	Denmark	Women who had a tubal sterilization (1977–1993)	High	30	65,232	–	–	SIR	0.7	0.5	1	–	–	–	–
Rosenblatt KA ^15^	1997	1454	Case control	Seven countries	Older than 15	Moderate	35	377	101	1077	OR	–	–	–	1.26	0.79	2	Parity and age, hospital and date of diagnosis
Wernli KJ ^11^	2006	259,640	Cohort	China	Women employed in the textile industry	Moderate	59	–	147	–	HR	–	–	–	1.1	0.79	1.56	Age at baseline and reproductive category
Nagle CM ^6^	2008	1650	Case control	Australia	Cancer registries	Moderate	19	425	404	1225	OR	–	–	–	0.4	0.3	0.7	Age, education, parity and hormone contraceptive use
Iversen L ^18^	2007	5602	Cohort	United Kingdom	Women who were using oral contraceptives	High	1	2801	4	2801	–	–	–	–	–	–	–	–
Gaitskell K ^10^	2016	1,278,783	Cohort	United Kingdom	UK women	High	2018	206,233	8571	767,251	RR	–	–	–	0.98	0.93	1.03	Age, region, socioeconomic status, parity, age at first birth, hysterectomy, smoking, alcohol intake, physical activity, body mass index, and use of the oral, contraceptive pill or menopausal hormones
Winer I ^9^	2016	76,483	Cohort	USA	50 to 79 years, postmenopausal	High	192	14,499	945	61,984	HR	0.87	0.75	1.05	0.9	0.76	1.07	Age stratum at randomization and region
Falconer H ^2^	2018	5,385,186	Cohort	Switzerland	Swedish women	High	281	80,765	35,430	5,304,421	HR	–	–	–	0.73	0.65	0.83	Age, parity, calendar time and education status

*DOP* Date of publication, *SS* Sample size, *TL* Tubal ligation, *TES* Type of reported effect size, *ES* Effect size, *UL* Upper limit, *LL* Lower limit, *AES* Adjusted effect size, *OR* odds ratio, *SIR* Standardized incidence ratio, *RR* Risk ratio, *HR* Hazard ratio, *OC* Oral contraception

*Based on the Newcastle-Otawa scale

### Quantitative data synthesis

**Fig. 3:**
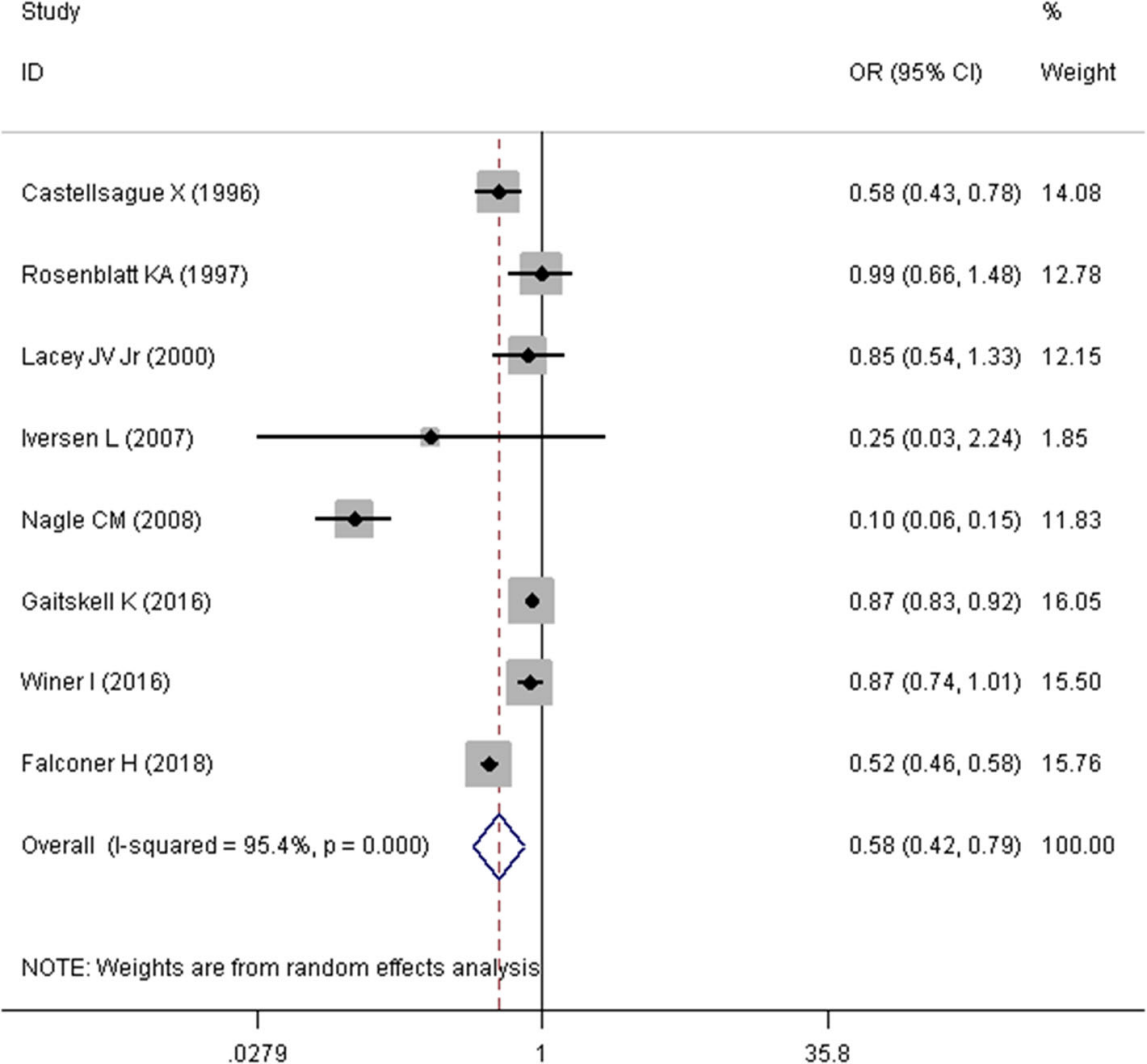
Forest plot describing the association between tubal ligation and endometrial cancer risk using raw numbers of the table’s cells

**Fig. 4:**
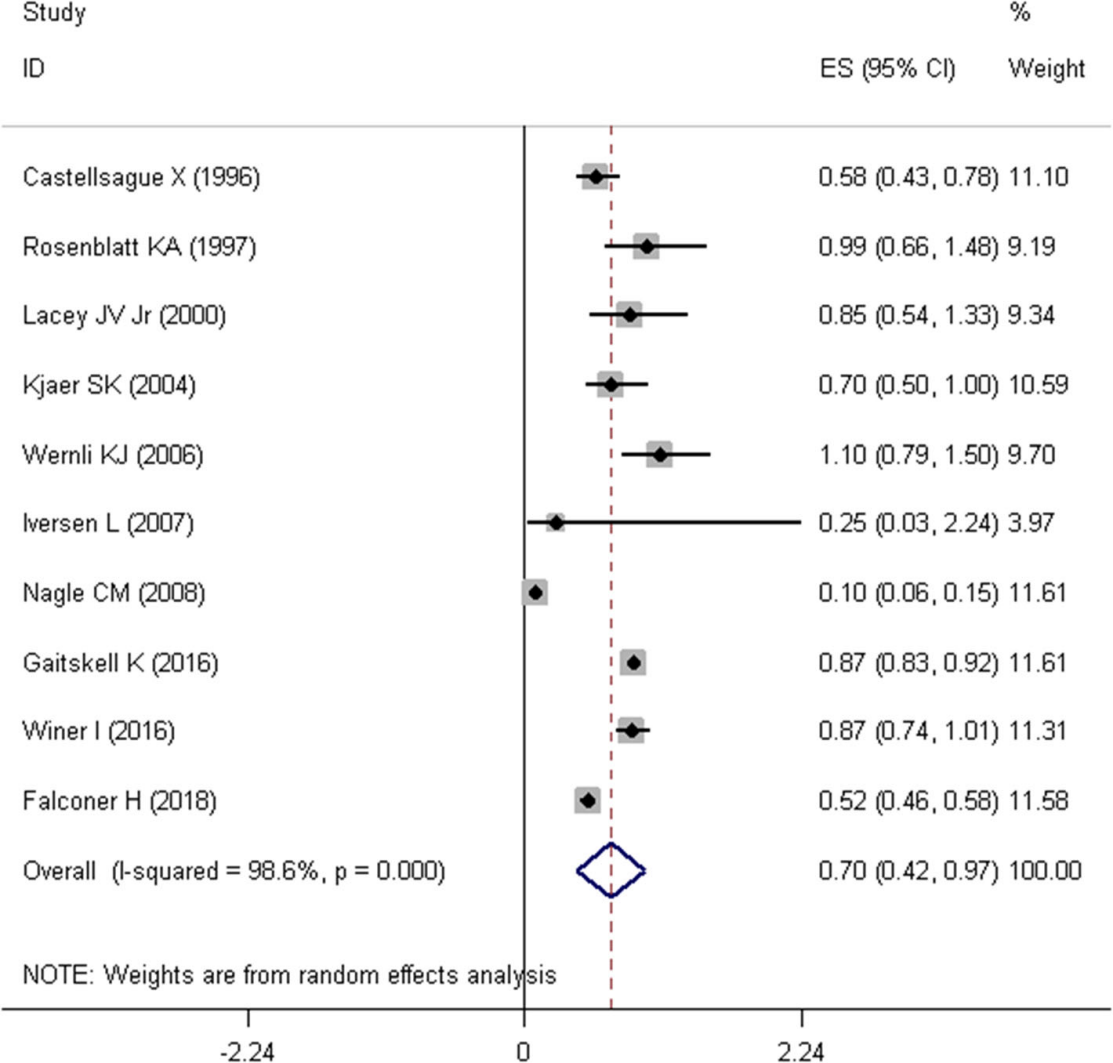
Forest plot describing the association between tubal ligation and endometrial cancer risk using reported effect sizes

**Fig. 5:**
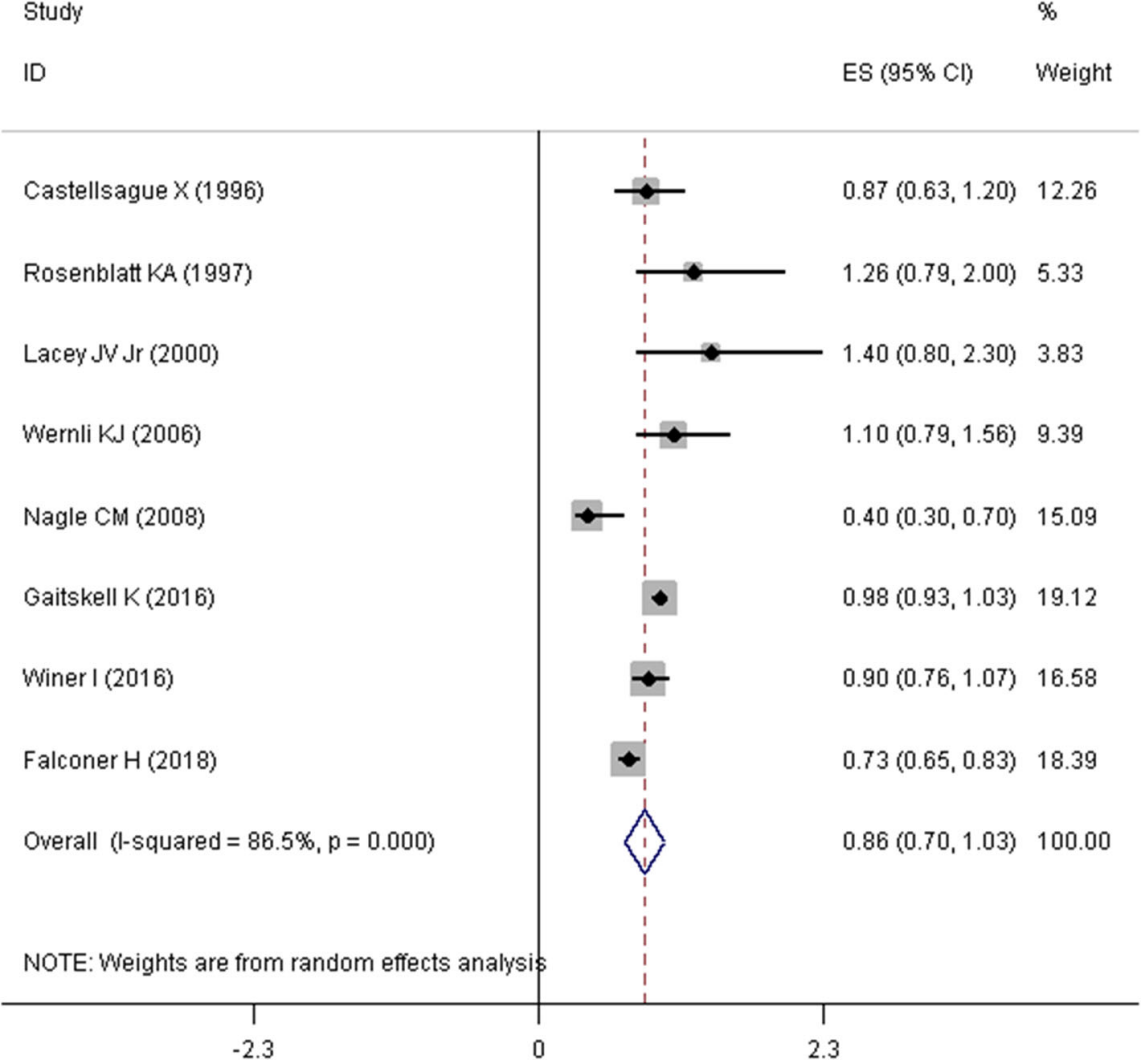
Forest plot describing the association between tubal ligation and endometrial cancer risk using adjusted effect size

Mantel–Haenszel approach was used to estimate the SOR and its corresponding 95% confidence interval (95% CI). Due to presence of heterogeneity problem, a random effects model was used to pool the OR of the studies. Firstly, we pooled the raw numbers of cells (i.e. a, b, c, and d) in tables of eight studies that reported these numbers. As it was presented in Fig. 3, the summary estimate of odds ratio (SOR) in this meta-analysis suggested that tubal ligation was significantly associated with a lower risk of endometrial cancer (SOR = 0.577, 95%CI = 0.420–0.792, I^2^ = 95.4%). Two other studies only reported the standardized incidence ratio (SIR) [25] and hazard ratio (HR) [17]. Secondly, given the rare nature of endometrial cancer (less than 5%), SIRs, HRs, and ORs were considered as comparable measures of disease risk [27, 28]. Therefore, we pooled the ten studies and estimated the relationship between tubal ligation and endometrial cancer risk. As depicted in Fig. 4, SOR of ten studies revealed that tubal ligation was significantly associated with a lower risk of endometrial cancer risk (SOR = 0.696, 95% CI = 0.425–0.966, I^2^ = 98.6%). Thirdly, eight studies [2, 12, 15–17, 22–24] in which adjusted effect size (i.e. OR, RR, SIR, and HR) was reported were pooled together (Fig. 5). Meta-analysis of these studies displayed that the summary estimate of adjusted odds ratio (SAOR) of tubal ligation and endometrial cancer risk tended to be significant (SAOR = 0.862, 95% CI = 0.698–1.026, I^2^ = 86.5%).

### Heterogeneity and meta-regression

There was huge heterogeneity (heterogeneity chi-squared = 152.30, d.f. = 7, *p* < 0.001, I-squared (variation in OR attributable to heterogeneity) = 95.4%, estimate of between-study variance Tau-squared = 0.1627) in eight studies (with raw numbers) that were pooled together. There was also considerable heterogeneity (heterogeneity chi-squared = 623.04, d.f. = 9, *p* < 0.001, I-squared = 98.6% and estimate of between-study variance Tau-squared = 0.1639) in 10 studies that their ORs were pooled together. Also, there was sizeable heterogeneity among studies for which we pooled their adjusted effect sizes (heterogeneity chi-squared = 51.68, d.f. = 7, *p* < 0.001, I-squared = 86.5%, estimate of between-study variance Tau-squared = 0.0359). As a result of these revealed heterogeneities, random effect model was used to pool the effect sizes. In addition, meta-regression method was also used to find the source of heterogeneity.

The results of meta-regression ruled out the involvement of sample size (less and more than 10,000) (*p* = 0.571), study design (cohort and case-control) (*p* = 0.616), date of publication (before and after 2008) (*p* = 0.613) and study quality (high and moderate) (*p* = 0.569) on observed heterogeneity among studies.

#### Subgroup analysis

As showed in Table 2, sub-group analysis was performed on variables of sample size, study design, data of publication, and study quality. The results showed that even subgroup analysis did not reduce the size of heterogeneity and there was no heterogeneity only in the studies published between 1996 and 2007. Therefore, a fixed effect model was used to estimate SOR in this subgroup (SOR = 0.71, 95% CI = 0.57–0.87, I^2^ = 48.8%).

**Table 2:** Summary of meta-analysis results and subgroups analysis

Groups	Test of association				Heterogeneity		
	OR(95% CI)	*P* value	Model	Z	Chi square	*P* value	I square
study design							
Cohort	0.72(0.51–1.00)	0.053	Random	1.94	67.04	0.001	95.5%
Case control	0.47(0.18–1.21)	0.116	Random	1.57	68.32	0.001	95.6%
Date of publication							
1996–2007	0.71(0.57–0.87)	0.001	Fixed	3.22	5.86	0.119	48.8%
2008–2018	0.48(0.31–0.80)	0.001	Random	3.24	145.62	0.001	97.9%
Sample size							
Less than 10,000	0.44(0.18–1.07)	0.075	Random	1.78	65.91	0.001	97.0%
More than 10,000	0.73(0.52–1.03)	0.071	Random	1.81	68.70	0.001	94.2%
Study quality							
High	0.71(0.54–0.92)	0.011	Random	2.54	72.00	0.001	93.1%
Moderate	0.31(0.03–3.34)	0.333	Random	0.97	58.49	0.001	98.3%
Overall	0.58(0.42–0.80)	0.001	Random	3.40	152.30	0.001	95.4%

### Publication bias and sensitivity analysis

Publication bias was checked for the relationship between tubal ligation and endometrial cancer risk and no evidence for publication bias was found (Begg’s test statistic; − 0.74, *P* = 0.46). After removal of Iversen et al. study [26], because of low number of endometrial cancer cases in each group, a sensitivity analysis was performed. SOR was also re-calculated in order to assess the extent to which SOR was influenced by Iversen et al. study.. We concluded that after removal of Iversen et al. study [26], SOR changed notably from 0.577 (95% CI = 0.420–0.792) to 0.706 (95% CI = 0.524–0.888). Newcastle-Ottawa assessment scale also showed that 7 and 3 studies had high and moderate quality, respectively.

## Discussion

A number of studies have reported controversial findings regarding the relationship between tubal ligation and endometrial cancer risk. The most important objective of this meta-analysis of studied was to document the association of tubal ligation and endometrial cancer risk. To our knowledge, this is the first meta-analysis of tubal ligation and endometrial cancer risk. The findings of this systematic review and meta-analysis suggest that tubal ligation is significantly related with a reduced risk of endometrial cancer (approximately 42% reduction in risk). Moreover, risk of endometrial cancer in women with tubal ligation is 0.577 times lower that that of women without tubal ligation (SOR = 0.577, 95% CI = 0.420–0.792). A considerable protective effect was also revealed in some subgroups of studies. In case of adjusted analysis, the results of studies were adjusted for various confounder variables. List of these variables for each study is shown in Table 1. However, following a meta-analysis of adjusted results, there was a 14% reduction in the risk of endometrial cancer for women with a tubal litigation history.

Wernli et al. [17] suggested no significant relationship between tubal ligation and endometrial cancer risk (HR 1.11, 95% CI: 0.79–1.56) among Chinese women. Similar to Wernli et al. [17] study, Gaitskell et al. [16] in the Million Women Study revealed of no significant relationship (RR: 0.98, 95% CI: 0.93, 1.03). Contrary to these two studies, Falconer et al. [2] study in a Swedish population-based cohort study in 2018 revealed a significant relationship between tubal ligation and endometrial cancer risk (HR: 0.73, 95% CI: 0.65–0.83). This contradiction in findings can be due to different adjusted variables in each study. Variables of Wernli et al. study were adjusted for “age at baseline and reproductive category”. Variables of Gaitskell et al. study were adjusted for “age, region, socioeconomic status, parity, age at first birth, hysterectomy, smoking, alcohol intake, physical activity, body mass index, and use of the oral contraceptive pill or menopausal hormones”. Finally, Variables in Falconer et al. study were adjusted for “age, parity, calendar time, and education status”.

The relationship of tubal ligation and lower risk of endometrial cancer has been concluded in previous studies [2, 12, 25], but there are studies that have not reported this association as well [15, 17, 22–24]. Based on the results of our study, tubal ligation appears to be associated with a reduced risk of endometrial cancer and SAOR tends to be significant (SAOR = 0.862, 95% CI = 0.698–1.026).

Although the exact mechanism of tubal ligation and reduced risk of gynecological cancer is vague, there are some theories that have aimed to explain the relationship. In particular, one theory holds that occlusion of the fallopian tubes after tubal litigation acts as a physical obstacle and can obstruct passage of exfoliative cells (carcinogenic talc transportation or other agents) from the external genitalia and vagina into the peritoneal cavity, ovary, and fallopian tubes [14, 16, 29]. Also, results of a meta-analysis by Rice et al. [30] suggest that hysterectomy and tubal ligation are related with reduced risk of ovarian cancer as well. Moreover, in a meta-analysis by Cibula et al. [31], the researchers revealed a significant reduction of ovarian cancer risk in women with a history of tubal ligation. Our study findings are also consistent with findings of Wang et al. [32] on epithelial ovarian cancer in which they concluded that tubal ligation is related with a lower risk of epithelial ovarian cancer (OR = 0.70, 95% CI = 0.60–0.81).

Although only 5% of endometrial cancer cases occur before age 40 [33], chance of fertility should not be ruled out for these women. Management of endometrial cancer, especially in its early stages (stage I and IA, and G1 or G2), progestin therapy, and a conservative therapeutic regime are recommended to preserve the chance of fertility [34, 35]. In addition to progestin therapy, fertility-sparing surgery can be also used as an appropriate option for patients at childbearing age with early stages of endometrial cancer [34].

The results of analysis in subgroups showed that the association between tubal ligation and endometrial cancer risk was significant in cohort studies as well as in studies with high quality Compared to cohort studies; however, case-control studies usually have less sample size and therefore less power to detect the relationship.

One of the main concerns in meta-analysis studies is the presence of confounding variables and their inappropriate adjustment. In this study, we extracted the adjusted effect sizes and performed a meta-analysis on them. Although different confounding variables had been adjusted in individual studies, this analysis, to some extent, reduced the existing concerns. The results of SAOR were consistent with the unadjusted results and supported the protective effect of tubal ligation. However, standardized incidence ratios, hazard ratios, odds ratios, and risk ratios were considered as comparable measures of risk in this study [36]. As incidence of endometrial cancer is very rare (say less than 5%), equal consideration of these effect sizes did not cause serious problems.

Following from previous studies, databases of PubMed/Medline, Web of Science, Scopus, and Embase were searched to find the relevant studies. Google Scholar and the references of relevant full texts were also searched to prevent from missing the relevant studies. Therefore, it seems that probability of missing the eligible studies was minimized. Nonetheless, it should be noted that only English language full-texts were retrieved and there may be some non-English articles that were missed.

Begg’s test suggested that there was no publication bias, but heterogeneity among the studies was meaningful. We tried to find the source of this heterogeneity by running a meta-regression, but the results of meta-regression rejected the role of sample size, study design, data of publication, and study quality on heterogeneity among the studies. Possibly, a part of this heterogeneity is attributable to presence of different populations in the studies. For instance, Winer et al. [15] conducted a study on postmenopausal women aged 50 to 79 years old, while the study of Karin et al. [23] was on women who were over 15 years old.

As it was mentioned in the methods section all kinds of tubal ligation were pooled together in our analysis. But, it should be noted that there are some differences between mono-lateral and bi-lateral salpingectomy, tubal coagulation with or without cut, and tubal ligation by stitches with or without cut. However, since the kind of tubal ligation was not specified in the studies, there was no possibility for us to conduct a sub-group analysis. Nonetheless, it is recommended to conduct a study in future to check the association of different types of female sterilization with endometrial cancer.

## Conclusions

In summary, this meta-analysis revealed a protective effect for tubal ligation against endometrial cancer risk (roughly 42% reduction in risk). To make this finding more implementable, however, additional longitudinal studies with relatively large sample size and long follow-up designs are necessary. It is also recommended that future studies should aim to reveal the mechanism of this relationship as well.

## Data Availability

All data generated or analysed during this study are included in this published article [and its supplementary information files].

## Abbreviations

CI: Confidence Interval
IUD: Intrauterine Device
MeSH: Medical Subject Headings
OR: Odds Ratio
PRISMA: Preferred Reporting Items for Systematic Review and Meta-Analysis
SAOR: Summary Adjusted Odds Ratio
SOR: Summary Odds Ratio
